# Responses of photosynthesis and chlorophyll fluorescence during light induction in different seedling ages of *Mahonia oiwakensis*

**DOI:** 10.1186/s40529-023-00369-w

**Published:** 2023-03-09

**Authors:** Chung-I. Chen, Kuan-Hung Lin, Tzu-Chao Lin, Meng-Yuan Huang, Yung-Chih Chen, Chau-Ching Huang, Ching-Wen Wang

**Affiliations:** 1 Department of Forestry, National Pingtung University of Science and Technology, Pingtung, 91201 Taiwan; 2 Department of Horticulture and Biotechnology, Chinese Culture University, Taipei, 11114 Taiwan; 3 Endemic Species Research Institute, Jiji Township, No.1, Minsheng E. Rd, Nantou County, 55244 Taiwan; 4 Department of Life Sciences and Innovation and Development Center of Sustainable Agriculture, National Chung Hsing University, Taichung, 40227 Taiwan; 5 College of Bio-Resources and Agriculture, The Experimental Forest, National Taiwan University, Nantou, 557 Taiwan

**Keywords:** Berberidaceae, Chlorophyll fluorescence, Light intensity, *Mahonia*, Photosynthesis efficiency, Energy quenching, Photo-inhibition, Photosystem II

## Abstract

**Background:**

The aim of this study was to determine the actual state of the photosynthetic apparatus and exhibit distinguishable differences in the chlorophyll fluorescence (ChlF) components in different seedling ages of *M. oiwakensis* plants subjected to different light intensity (LI). Potted 6-month-old greenhouse seedlings and field collected 2.4-year-old seedlings with 5 cm heights were selected and randomly separated into seven groups for photosynthesis measurements illuminated with 50, 100 (assigned as low LI), 300, 500, 1,000 (as moderate LI), 1,500 and 2,000 (as high LI) μmol m^–2^ s^–1^ photosynthetic photon flux density (PPFD) treatments.

**Results:**

n 6-month-old seedlings, as LI increased from 50 to 2,000 PPFD, the values of non-photochemical quenching and photo-inhibitory quenching (qI) increased but potential quantum efficiency of PSII (Fv/Fm) and photochemical efficiency of photosystem II (ΦPSII) values decreased. High electron transport rate and percentage of actual PSII efficiency by Fv/Fm values were observed in 2.4-year-old seedlings at high LI conditions. Furthermore, higher ΦPSII was detected under low LI conditions, with lower energy-dependent quenching (qE) and qI values and photo-inhibition % decreased as well. However, qE and qI increased as ΦPSII decreased and photo-inhibition% increased under high LI treatments.

**Conclusions:**

These results could be useful for predicting the changes in growth and distribution of *Mahonia* species grown in controlled environments and open fields with various combinations of varying light illuminations, and ecological monitoring of their restoration and habitat creation is important for provenance conservation and helps to formulate better conservation strategies for the seedlings.

**Supplementary Information:**

The online version contains supplementary material available at 10.1186/s40529-023-00369-w.

## Introduction

The *Mahonia* genus, a member of the Berberidaceae family, contains more than 60 species and is native to Asia and America (Jaca and Mkhize 2018). Many *Mahonia* species are folk medicinal plants, possess antibacterial, antifungal, and analgesic properties (Rohrer et al. 2017; Liu et al. 2020). *Mahonia oiwakensis* Hayata, an endemic species, is distributed in the central mountain range of Taiwan at an altitude of 1200 ~ 2,600 m. Worldwide and Taiwanese conservation assessment systems have declared it to be vulnerable and endangered, respectively, among their threatened categories because of more common in collapsed land, felled land, and in the late stage of secondary vegetation succession (International Union for Conservation of Nature 2001; Editorial Committee of the Red List of Taiwan Plants 2017). Since global warming severely affects biodiversity, the distribution and survival of organisms are closely related to climate, and deteriorating climatic conditions lead to the disappearance of some species (Schloss et al. 2012; Randall and van Woesik 2015). Studies have shown that high-altitude plants are particularly sensitive to climate changes and more vulnerable than other species (Dirnböck et al. 2011; Dullinger et al. 2012), including alpine plants in Taiwan (Chou et al. 2011). Therefore, understanding the ecological habits of *M. oiwakensis*, such as light adaptation range, is important for provenance conservation and helps to formulate better conservation strategies.

Light is an environmental signal, inducing chlorophyll (Chl) biosynthesis, and changes in light irradiance evoke variable photosynthetic responses. The photosynthetic induction process occurs when a leaf in the shade is suddenly exposed to a high LI, and the photosynthetic rate gradually reaches its maximum value in several minutes to an hour depending on the species, ecological habitat, and other environmental factors (Montgomery and Givnish 2008). Thus, eco-physiological responses to excess sunlight vary among different species or even during the plant life cycle, and may significantly affect the survival rate and spatial distribution of *M. oiwakensis* plants (Wong et al. 2014). In full sun-exposed habitats, leaves often absorb considerably more photons than can be utilized, the excess absorbed energy often resulting in a reduction of the photochemical efficiency of photosystem II (ΦPSII) in plants. A relatively high density of young *M. oiwakensis* seedlings grow slowly beneath the canopy of coniferous forests and rapidly in wide forest gaps and forest margins lacking grass competition, thereby exhibiting the characteristics of both light-demanding and shade-tolerant species (Lu and Yang 1996). Insufficient light can limit photosynthesis, causing reductions in net carbon gains and plant growth. Conversely, under high irradiance, excess light may result in photo-inhibition, which is characterized by a loss of PSII activity and a light-dependent reduction in the fundamental quantum yield of photosynthesis, requiring the dissipation of excess excitation energy (Portela et al. 2019). Thus, non-photochemical quenching (NPQ) plays an important role in photoprotection because it quenches excess energy and dissipates it safely as heat (Murchie and Niyogi 2010). Moreover, plants exposed to strong light also present decreases or adjustments in their leaf photosynthetic pigment concentration, providing an important photo-protective mechanism (Souza et al. 2017).

Chlorophyll fluorescence (ChlF) is a noninvasive technique offering highly accurate measurements that illustrate the function of the photosynthetic apparatus in plants, and fluorescence is often used in physiological studies to investigate a plant’s response to various environmental stresses in controlled environments and in the field (Kałuzewicz et al. 2018). ChlF values reflect photosynthetic potential and the potential for photochemical dissipation, and they demonstrate the percentage of PSII that is open and its effectiveness in capturing photo energy from light-harvesting complexes (LHC), and the subsequent transfer of quanta (Moya et al. 2019). Non-photochemical quenching (NPQ) in ChlF parameters can be divided into photo-protection and photo-inhibition, among which photo-protection makes an important contribution to the dissipation of excess energy in the plant photosynthetic system (Müller et al. 2001; Murchie and Niyogi 2010). NPQ can be subdivided into several components according to dark recovery after illumination. The fastest and most important component of NPQ is qE (energy-dependent quenching) (Müller et al. 2001; Johnson and Ruban 2011). This is closely related to the following three items: (1) changes in thylakoid membrane proton gradient (ΔpH), (2) the total amount of pigment involved in the xanthophyll cycle, and (3) the existence of the subunit PsbS in PSII (Müller et al. 2001; Lavaud and Lepetit 2013). Moreover, Nilkens et al. (2010) reported that after the completion of reaction in the subunit PsbS, zeaxanthin (Z) is combined with PsbS to dissipate H^+^, thus, the part after the reaction of qE is called qZ (zeaxanthin-dependent quenching). The second component of NPQ is qT (phosphorylation shift-dependent quenching), which shows the phosphorylation shift of the light-harvesting complex (LHC) II between PSII and PSI (Quick and Stitt 1989). The slowest reaction component of NPQ is qI (photoinhibitory quenching), which is related to photo-inhibition or slow reversible recovery of the PSII reaction center (Müller et al. 2001). Consequently, parameters, such as qI, qE and qZ + qT, can accurately evaluate the photosynthetic physiological adaptability of plants to various stresses (Lai et al. 2022; Wang et al. 2022). However, few studies have described their eco-physiological responses under controlled light conditions, and the function of the photosynthetic apparatus has not yet been examined with respect to the occurrence of ChlF indicators in *Mahonia* leaves under field conditions to explain the development and distribution of *Mahonia* species. Efforts to gain an understanding of the photosynthetic characteristics of *Mahonia* leaves can benefit field cultivation management. Therefore, it is urgent to regulate and prioritize it for management and add it to the list of nationally endangered species under surveillance for potential eradication or containment targeting.

In the present study, we analyzed ChlF parameters in various seedling ages of *M. oiwakensis* populations from the nursery and field to understand whether those young individuals could (1) acclimate to light conditions and have higher physiological plasticity in LI and presentation of shade-tolerant characteristics, and (2) predict the impact of anthropogenic climate change on plant performance and distribution. It is essential to assess how *M. oiwakensis* plants respond to altered growth light conditions. Therefore, we hypothesized that the mechanisms of the capture, transfer, and dissipation of excitation energy could be detected by ChlF measurements in different seedling ages of *Mahonia* leaves in response to varying LI, and *Mahonia* leaves would exhibit strong LI adjustment of photosynthesis. Our study of *M. oiwakensis* not only recognizes their ecological distinctness, but highlights their critical conservation status. In addition, the relationships of ChlF indices can be used for eco-physiological research in *Mahonia* species, and these parameters can be considered selection indices for examining the growth of *Mahonia* species grown under artificial light illuminations. The precise management of ChlF parameters in response to the various LI may maximize the economic efficiency of the growth and development potential of *Mahonia* species grown in controlled environments. Thus, it is of great significance for conservation of provenance, ex situ conservation and propagation of *Mahonia* seedlings.

## Material and methods

### Plant materials and cultural practices

#### Collections of 6-month-old seedlings from the greenhouse

*M. oiwakensis* Hayata (*Alishan mahonia*) propagules were collected from the Nantou Mountain area (23°38′54.7"N 120°47′40.6"E), and have been grown and maintained in our greenhouse**s** at the Endemic Species Research Institute, Nantou, Taiwan (23°49′43.0" N, 120°48′04.7" E). The seeds of *M. oiwakensis* Hayata were planted and grown in round plastic pots (7 cm diameter and 10 cm depth) containing commercial potting soil with a substrate mixture of loam: sand = 4:1, and placed in the above-mentioned greenhouses at 25 °C day/night temperatures, relative humidity of 80%, and 50 ~ 200 μmol·m^−2^·s^−1^ photosynthetic photon flux density (PPFD) during February to August 2021. Plants were watered once a week, an optimal amount of a compound water-soluble fertilizer solution (N:P_2_O_5_:K_2_O, 20:20:20; Scott, Marysville, OH, USA) applied weekly at 1 g L^−1^, and allowed to grow for 6 months (Additional file 1: Fig. S1A) before collecting the ChlF measurements described below.

#### Collections of the 2.4-year-old seedlings in the field

For the field experiments, five 2.4-year-old seedlings of *M. oiwakensis* Hayata (*Alishan mahonia*) with 5 cm heights were selected in our outpost station at the Hehuan forest mountain area of Nantou (24°06′01.2"N 121°11′33.3"E) where we have been monitoring *Mahonia* species for 3 years (Additional file 1: Fig. S1B). Plants were studied in the field, and vegetation and habitat types were recorded for the sites where *M. oiwakensis* Hayata was collected; however, we were not able to find 6-month-old seedlings in the field sites during the study period. The study site has a humid subtropical climate, with a mean annual rainfall of 2,000 mm, mean annual air temperature of 16.5 ℃, and a mean LI of less than 100 μmol·m^−2^·s^−1^ PPFD, as recorded from Jan to Dec 2021 (Additional file 1: Fig. S2). The soil at the Hehuan station is a typical Andosol in which the texture of the upper surface is sandy loam.

### Determination of ChlF variables under a fixed light intensity

The above-mentioned potted 6-month-old seedlings with 5 cm heights were selected and moved to a dark room overnight at 25 ^◦^C. Five plants (one plant in a pot) with fully open top leaves were used for ChlF measurements. In addition, the upper fully expanded leaves of 2.4-year-old seedlings were collected from July to August 2021 for the following LI experiments. Five plants (one leaf per plant) per light treatment were used for ChlF measurements. The surfaces of the leaves were illuminated with 50, 100, 300, 500, 1000, 1500, and 2000 μmol m^–2^ s^–1^ PPFD using a portable pulse amplitude-modulated fluorometer (PAM-2000, HeinzWalz, Effeltrich, Germany). Dark-adapted plants were exposed to light stepwise from low to high levels of PPFD, and ChlF parameters were measured during 60 min of irradiation and dark adaptation for 30 min. individual data points were recorded at 2 min intervals over a 90 min period, followed by calculating the parameters below. Seven gradients of photometry were used to measure two ages of *M. oiwakensis,* but more detailed light adaptation assessments were performed due to little differences in parameters observed in the light curve (Additional file 1: Fig. S3).

The potential quantum efficiency of PSII (Fv/Fm) was calculated from (Fm - Fo) / Fm (Demmig-Adams et al. 1996). The actual PSII efficiency (ΔF / Fm’) is the effective quantum yield of linear electron flux through PSII, and used to express the ability of PSII to perform photochemistry. Values of the minimal ChlF (Fo) and maximal ChlF (Fm) of dark-adapted samples were determined using modulated irradiation of a weak light-emitting diode beam (measuring light) and saturating pulse, respectively. Fm’ is the maximal fluorescence during illumination determined by applying a saturating flash. Measured leaves were dark-adapted for 30 min before performing light-inducing runs. The photochemical ΦPSII was calculated as (Fm’ - Ft)/Fm’, where Ft is the steady-state fluorescence at each PPFD level (Maxwell and Johnson 2000). Furthermore, the degree of photo-inhibition is calculated as 100% minus the relative value of F_v_*/*F_m_ after 30 min of dark adaptation, where the F_v_*/*F_m_ value of the same leaves before illumination is considered to be 100%. The apparent rate of the photosynthetic electron transport rate (ETR) of PSII was obtained as ETR = ΔF*/*F_m_′ × PPFD × 0.5 × α, where the factor 0.5 assumes equal excitation of both PSII and PSI; α is leaf absorption, and we used the mean “default” value of 0.84 for green leaves (Björkman and Demmig 1987; Lin et al. 2020). The following effective quantum yields were measured using the instant light-response curve program. From these data, several parameters can be computed based on modulated fluorescence kinetics. Degree of photoinhibition (photo-inhibition %) = 100%—relative value of Fv/Fm after 30 min of dark adaptation (Fv/Fm value of same leaves before illumination as 100%). The NPQ coefficient and its components: NPQ = (Fm—Fm’) / Fm’ (Müller et al. 2001; Weng et al. 2011). Energy-dependent quenching (qE) of NPQ is a mean of rapidly quenching energy, which is calculated as (Fm dark (2 min)—Fm’60 min) / Fm’60 min = (F_m_D_2_—F_m60_′) / F_m60_′ (Johnson and Ruban 2011). However, photo-inhibitory quenching (qI) is NPQ due to decreased CO_2_ fixation, which is calculated as [Fm—Fm dark (60 min)] / Fm’30 min = (F_m_*—*F_m_D_30_) / F_m60_′ (Müller et al. 2001). In addition, the part after the reaction of qE is (qZ + qT), and calculated as (F_m_D_30—_F_m_D_2_*) /* F_m60_′ (Maxwell and Johnson 2000; Nilkens et al. 2010)*.* The F_m60_′ is the maximum fluorescence value of leaves at 60 min of light exposure. Both F_m_D_2_ and F_m_D_30_ are the F_m_ values measured at 2 and 30 min, respectively, after dark recovery (Müller et al. 2001; Wang et al. 2022). Measurements were recorded with WinControl-3 software (Heinz Walz).

### Data analysis

All LI treatments were arranged in a completely randomized design, and all ChlF parameters were subjected to a single-factor analysis of variance (ANOVA) to determine whether a significant difference level of *p* ≤ 0.05 (using PASW Statistics 18 software (PASW 18, IBM, USA) existed between different treatments. Five leaves (one leaf per plant) were measured in each LI treatment (for a total of 35 plants), and data from each leaf represented one replicate in the statistical analyses. Regression analyses were used to examine relationships among qE, qI, and photo-inhibition %. Those model datasets were based on at least 15 leaves from each PPFD level. Several models were tested, including the linear regression models being selected for the interpretation of the relationship between ChlF parameters and PPFD. All models were evaluated for goodness of fit by the graphical analysis of residuals and by computing correlation coefficients at a significance level of p ≤ 0.05 for ChlF parameters. The linear regression model performance was most suitable.

## Results

In this study, the effects of the two seedling ages on *M. oiwakensis* during seven light intensities were monitored by measuring the changes in photosynthetic parameters, including ETR, NPQ, ФPSII, photo-inhibition, qE, qZ + qT, qE + qZ + qT and qI). Table 1 shows that all photosynthetic fluorescent parameters displayed significant differences (*p* < 0.01 and 0.05) in the main effects, except for ETR, ФPSII, and qZ + qT. Moreover, all fluorescent indices were significantly different (*p* < 0.0001) in interaction effects (A x L).

**Table 1:** Analysis of variance of the seedling age (A) and light intensity (L), and their interactions (A × L) on ETR, NPQ, ФPSII, photo-inhibition, qE, qZ + qT, qE + qZ + qT and qI of plant. Measurements were made at 25 °C under 50, 100, 300, 500, 1,000, 1,500, and 2,000 μmol m^−2^ s^−1^ PPFD

Parameter	Main effect			
	seedling age (A)		light intensity (L)	
	F and p value with significance			
	F	p	F	p
ETR	0.08	0.778^NS^	85.51	< 0.0001****
NPQ	4.91	0.005**	74.62	< 0.0001****
ФPSII	3.75	0.100^NS^	53.05	< 0.0001****
photo-inhibition	7.76	0.007**	26.19	< 0.0001****
qE	5.46	0.022*	38.25	< 0.0001****
qZ + qT	3.78	0.056^NS^	35.99	< 0.0001****
qE + qZ + qT	5.07	0.028*	85.64	< 0.0001****
qI	4.79	0.032*	53.27	< 0.0001****

Trait	Interaction effect	
	A x L	
	F and p value with significance	
	F	p
ETR	16.63	< 0.0001****
NPQ	57.89	< 0.0001****
ФPSII	30.12	< 0.0001****
photo-inhibition	21.62	< 0.0001****
qE	57.38	< 0.0001****
qZ + qT	17.82	< 0.0001****
qE + qZ + qT	33.98	< 0.0001****
qI	56.08	< 0.0001****

^*^p ≤ 0.05, ** p ≤ 0.01, **** p ≤ 0.0001,NS, Non-significant difference; n = 35 and 10 plants (replicates) for seedling age and LI, respectively, and n = 70 plants for interaction effect (A x L)

**Fig. 1:**
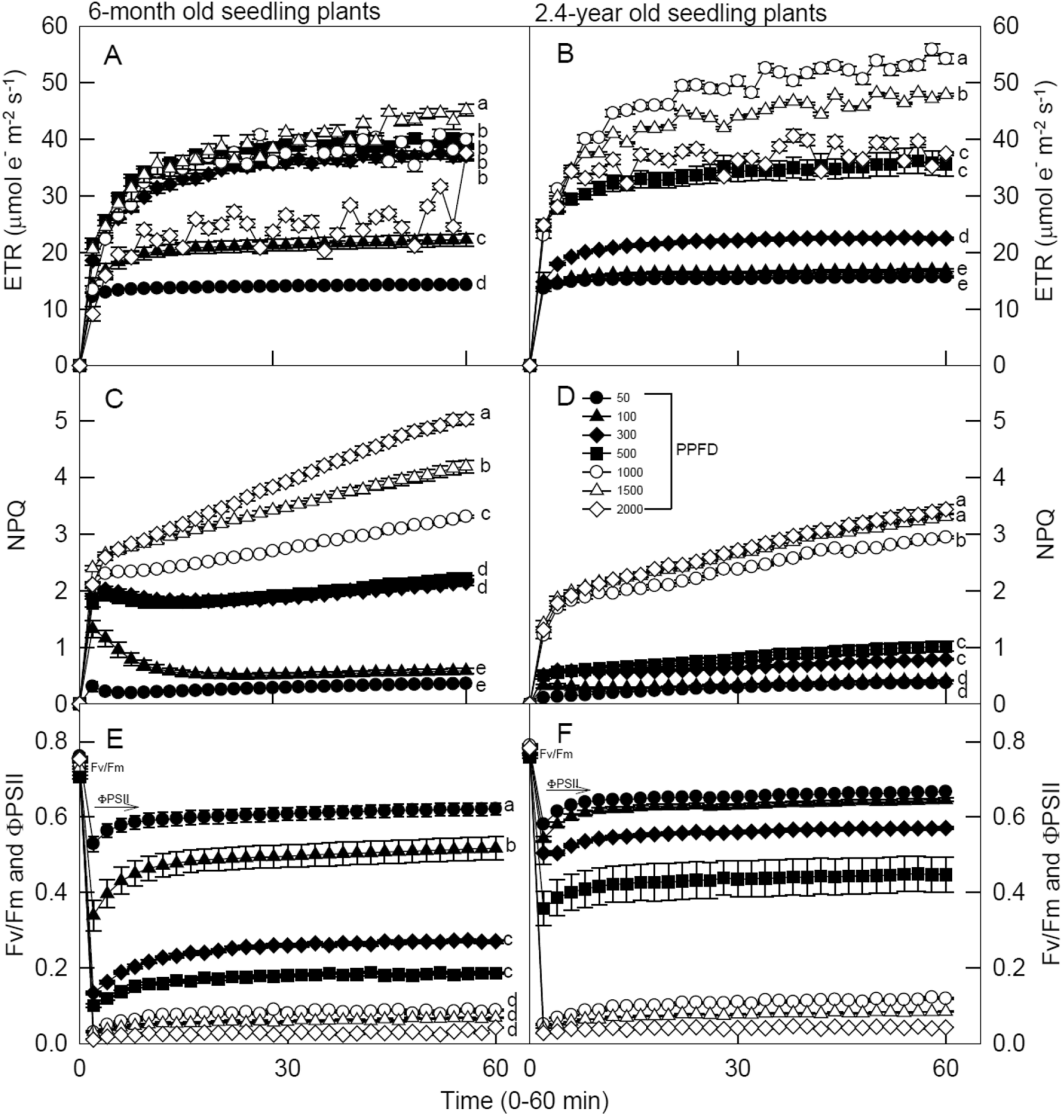
Time-course changes in electron transport rate (ETR), non-photochemical quenching (NPQ), and both Fv/Fm (unlit) and PSII efficiency (ФPSII, 2 ~ 60 min) for 6-month (panels **A**, **C**, and **E**) and 2.4-year (panels **B**, **D**, and **F**) old seedlings of *Mahonia oiwakensis*. During the 60-min of light induction, the measurements were made at 25 °C under 50, 100, 300, 500, 1,000, 1,500, and 2,000 μmol m^−2^ s.^−1^ photosynthetic photon flux density (PPFD). Vertical bars indicate standard errors, and each point represents the mean of 5 leaves. Different letters indicate significant differences in the Tukey's HSD analyses at seven light intensity treatments (*p* < 0.05)

Figure 1 shows the time-course changes in the photosynthetic light induction period of ETR, NPQ, and both Fv/Fm and ΦPSII for 6-month (Fig. 1A, C and E) and 2.4-year-old seedlings (Fig. 1B, D and F) of *Mahonia oiwakensis*. Measurements were initially obtained under dark followed by exposure to 50, 100 (low LI), 300, 500, 1000 (moderate LI), 1500, and 2000 (high LI) *μ*mol m^−2^ s^−1^ PPFD light induction for 60 min at 25 °C. When these overnight dark-adapted leaves were exposed to all light illuminations, the ETR of 6-month-old seedlings suddenly increased at the beginning of 2 min, but low LI treatments remained low (12 and 20 μmol m^−2^ s^−1^, respectively) as time passed, and no reduction of electron transport was detectable at longer illumination times compared to the other LI treatments that were gradually increased thereafter (> 30 μmol m^−2^ s^−1^) (Fig. 1A). In addition, ETR values under high LI conditions were significantly higher than in other LI treatments. A similar trend was observed in the ETR of 2.4-year-old seedlings, but the ETR value (> 50 μmol m^−2^ s^−1^) under L-1000 was significantly higher than other LI treatments (< 50 μmol m^−2^ s^−1^) (Fig. 1B). Figure 1 C and D display NPQ being sharply increased in all leaves in high LI treatments. NPQ values for all seedlings under high LI conditions were significantly higher (> 2) than under other LI treatments (< 2), and continued to linearly increase until 60 min. Furthermore, all NPQ values in 6-month-old seedlings were higher than in 2.4-year-old seedlings under all treatments, indicating that high LI limited 6-month-old seedling leaf growth and development, but that 2.4-year-old seedlings can be grown under a specific and optimal light intensity. Notably, the NPQ of 6-month-old seedlings under L-100 peaked (= 1.8) at 2 min, and then dropped remarkably (= 0.4) thereafter during photo-inhibitory processes (Fig. 1C).

**Fig. 2:**
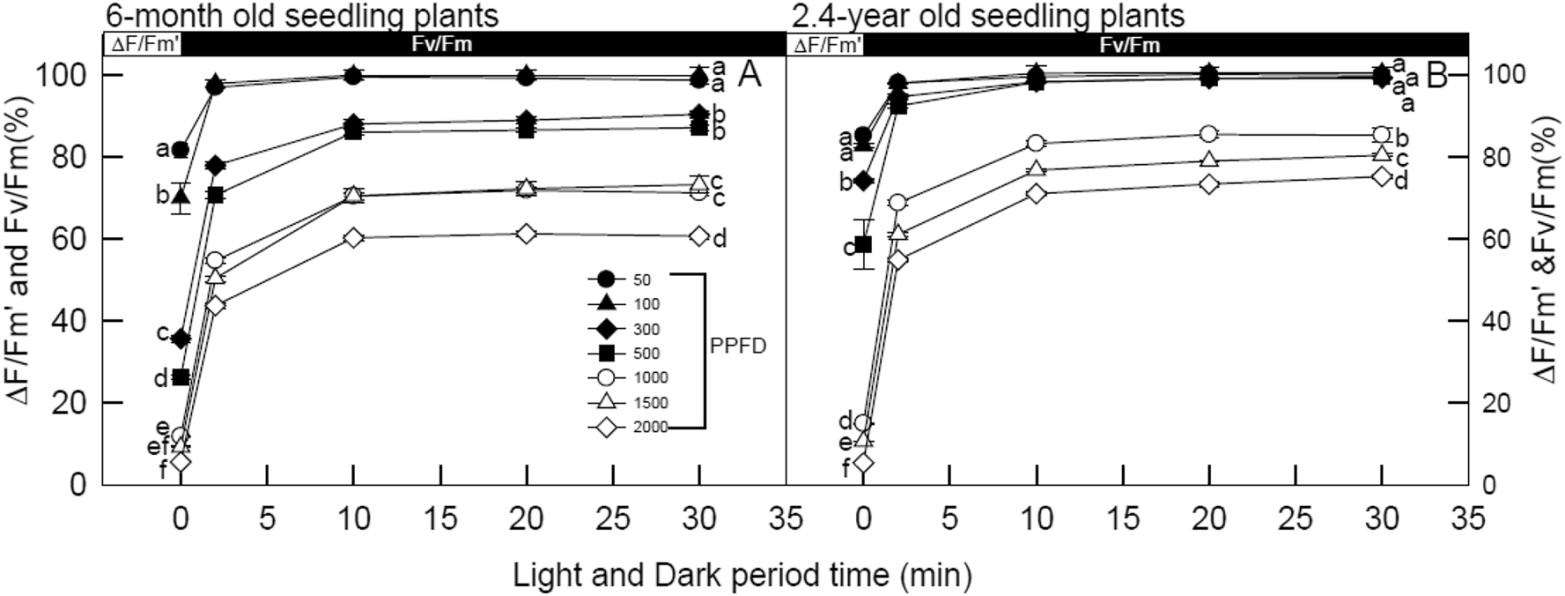
Relative value of actual PSII efficiency (ΔF/F’m) and potential quantum efficiency of PSII (Fv/Fm; Fv/Fm value of the same leaves before illumination set as 100%) obtained at artificial illumination of 60 min and subsequent dark recovery for 30 min (2 ~ 30 min dark) for 6-month (panel **A**) and 2.4-year (panel **B**) seedlings of *Mahonia oiwakensis* under 50, 100, 300, 500, 1,000, 1,500, and 2,000 μmol m^−2^ s.^−1^ PPFD at 25 °C. Vertical bars indicate standard errors, and each point represents the mean of 5 leaves. Different letters indicate significant differences in Tukey's HSD analyses for seven LI treatments (*p* < 0.05)

Figure 2 presents the time-course changes in the relative values of ΔF/F’m and Fv/Fm (Fv/Fm value of the same leaves before illumination as 100%) for 6-month and 2.4-year-old seedlings of *Mahonia oiwakensis*re under 50, 100, 300, 500, 1000, 1500, and 2000 μmol m^−2^ s^−1^ PPFD at 25 °C during 30 min darkness right after stopping illumination. Both ΔF/F’m and Fv/Fm (%) of all seedlings in all LI treatments rapidly increased in the dark right after stopping illumination, followed by gradually increasing and then remaining stable after 10 min of dark. The relative values of ΔF/F’m and Fv/Fm (%) of 6-month-old seedlings in high LI treatments recovered to 57.8% ~ 65.1% after 30 min of dark, which was significantly lower photo-protection compared to other LI treatments (79.6% ~ 99.8%). However, both ΔF/F’m and Fv/Fm (%) of 2.4-year-old seedlings in high LI treatments recovered to 70.8% ~ 82.7% after 30 min of darkness, but were still significantly lower than other LI treatments (98.7.6% ~ 99.9%), indicating that these seedlings can be grown under high LI conditions.

**Fig. 3:**
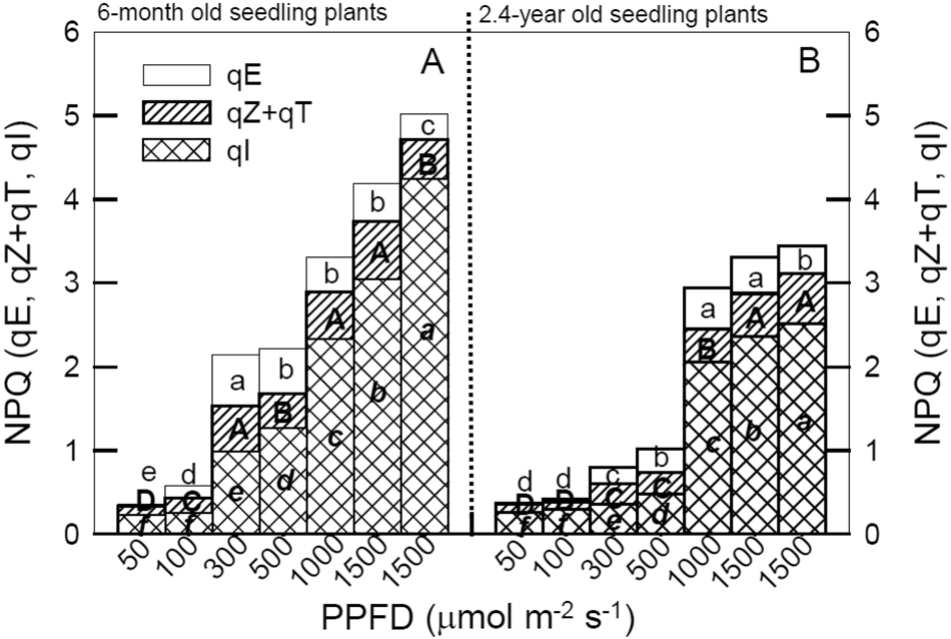
Composition of non-photochemical quenching (NPQ) in qE, qZ + qT, and qI fractions obtained at illumination for 60 min and after 30 min of dark period for 6-month (panel **A**) and 2.4-year (panel **B**) *Mahonia oiwakensis* under 50, 100, 300, 500, 1,000, 1,500, and 2,000 μmol m^−2^ s^−1^ PPFD at 25 °C. Different letters indicate significant differences in the Tukey's HSD analyses at seven LI treatments (*p* < 0.05). Each point represents the mean of 5 leaves

Figure 3 illustrates the fractions of NPQ, qZ + qT, qE, and qI obtained for 6-month and 2.4-year-old seedlings of *M. oiwakensis* at 50, 100, 300, 500, 1000, 1500, and 2000 μmol m^−2^ s^−1^ PPFD and after 30 min of dark. All seedlings at all LI substantially increased their NPQ, and the increase was not related to photo-protection (qE and qZ + qT) but to photo-inhibition (qI), and qI values increased as LI increased from 50 to 2000 μmol m^−2^ s^−1^ PPFD. The values of qZ + qT of all seedlings did not contribute remarkably to NPQ in LI treatments. All seedlings had significantly higher NPQ levels (ranged 3 ~ 5) in LI-1000, 1500, and 2000 compared to other LI treatments (< 2.2). Furthermore, 300, 500, 1000, 1500, and 2000 μmol m^−2^ s^−1^ treatments of 6-month-old seedlings produced a higher overall effect on NPQ than they did in 2.4-year-old seedlings, and their qI levels were also substantially increased.

**Fig. 4:**
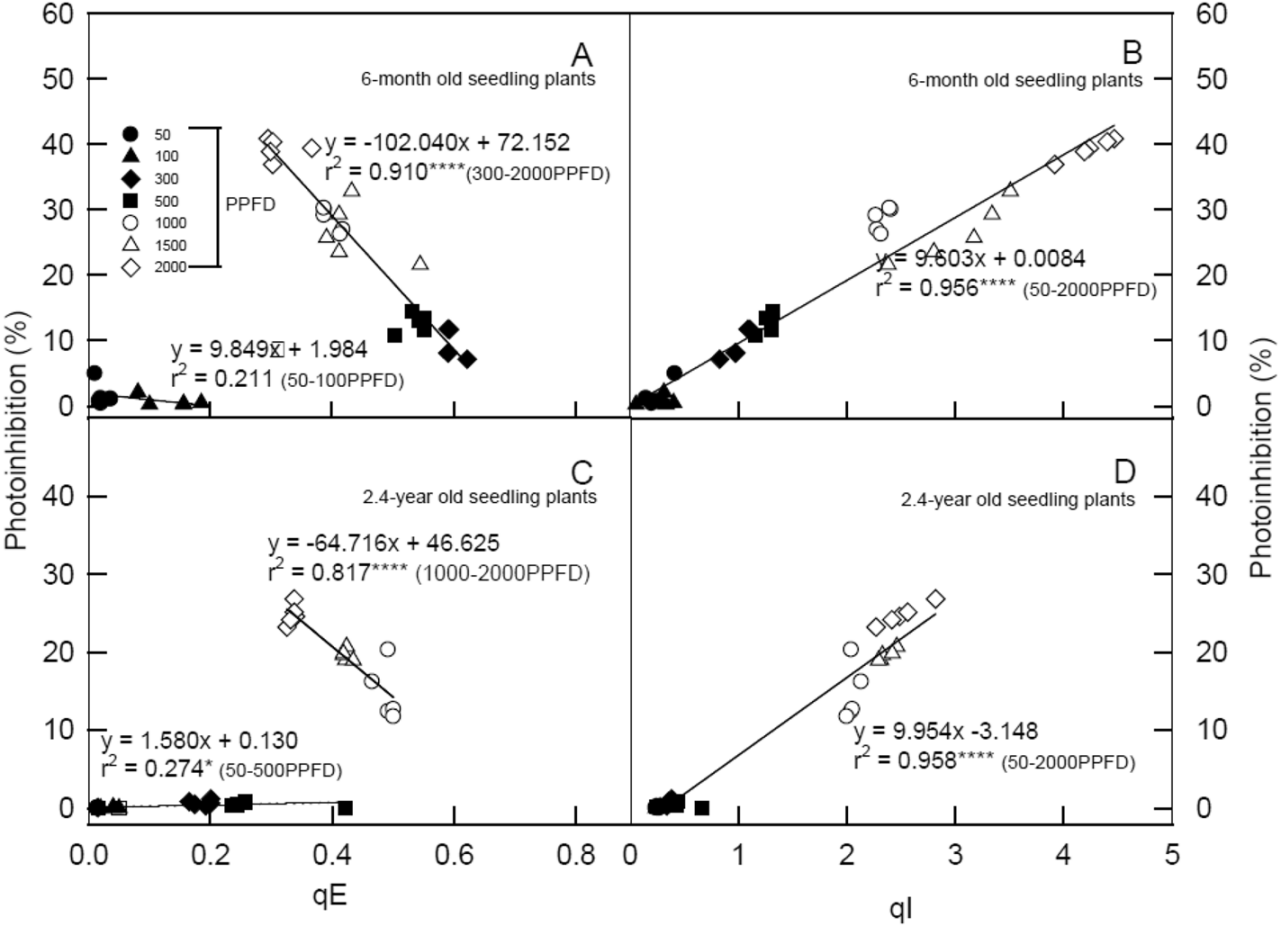
Correlations between photo-inhibition % to qE (panels **A** and **C**) and qI (panels **C** and **D**) in 6-month (panels **A** and **B**) and 2.4-year seedlings (panels **C** and **D**) of M. *oiwakensis* irradiated under 50, 100, 300, 500, 1,000, 1,500, and 2,000 μmol m^−2^ s^−1^ PPFD at 25 °C for 60 min. Each symbol represents the average of 5 leaves from one plant, and 5 plants were randomly selected from each light treatment. Each ChlF index was calculated using different seedling leaf data (n = 7) from model validation datasets. The determination coefficient (r.^2^) and significance of the regression are shown (*, **, ***, ****p < 0.05, 0.01, 0.001, 0.0001)

Figure 4 shows correlations between photo-inhibition% to qE and qI values from 6-month-old and 2.4-year-old seedlings under 50, 100, 300, 500, 1000, 1500, and 2000 μmol m^−2^ s^−1^ PPFD at 25 °C for 60 min. Significant and highly negative relationships were detected in photo-inhibition% and qE values under moderate and high LI conditions in 6-month-old and 2.4-year-old seedlings with r^2^ values of 0.971 (*p* < 0.01) and 0.978 (*p* < 0.05), respectively (Fig. 4A and C), implying that 6-month-old seedlings were more sensitive to high LI. Nevertheless, significant and highly positive correlations were observed between photo-inhibition% and qI values of 6-month-old and 2.4-year-old seedlings, with r^2^ values of 0.96 (*p* < 0.001) and 0.974 (*p* < 0.0001), respectively (Fig. 4B and D). The increased photo-inhibition% could have led to increases in qI value and decreases in qE value due to greater energy dissipation when plants were exposed to moderate and high LI conditions where photo-inhibition% in 6-month-old seedlings was higher than in 2.4-year-old seedlings.

## Discussion

Different seedling ages at varied LI treatments showed variable ChlF values from 0 ~ 60 min of light induction and 30 min of dark afterward. Seedlings developed at a given PPFD could be separated on the basis of ETR, NPQ, Fv/Fm, ΦPSII, ΔF/F’m%, and Fv/Fm% at a given LI. Generally, as LI increased from 50 to 2000 μmol m^−2^ s^−1^ PPFD, an increasing NPQ and decreasing Fv/Fm, ΦPSII, ΔF/F’m%, and Fv/Fm% were observed in all plants. The increase of NPQ includes both photoprotection and photo-inhibition. Within 2–4 min after the start of illumination, the plants showed a rapid increase in NPQ and a down-regulation of ΦPSII, which was a manifestation of photoprotective ability; therefore, both seedling ages exhibited high down-regulation of PSII efficiency (Zulfugarov et al. 2007). The studies on *Camptotheca acuminate* (Ma et al. 2015) and *Bletilla formosana* (Lai et al. 2022) also showed similar trends. When plants were exposed to high LI conditions, the excess energy in PSII increased, leading to increases in NPQ values. At this time, the increased NPQ is a light-suppressing portion, so the Fv/Fm value is in a state of decreasing. LI strongly affects the ETR and NPQ of plant leaves, and these values increased as LI increased except for 2.4-year-old seedlings at high LI conditions (Fig. 1B). The NPQ of 6-month-old seedling leaves was significantly higher than that of 2.4-year-old seedlings leaves at moderate and high LI conditions, indicating that 6-month-old seedlings have more non-photochemical quenching with greater damage from photo-oxidation compared to 2.4-year-seedlings. Fabricio et al. (2015) reported that the light tolerance of *Arabidopsis* plant increased with seedling age before 8 weeks old, but the light tolerance gradually declined after then. However, the differences in the NPQ of all plants were not significant in low LI conditions. Thus, the 6-month-old seedlings seemed adapted to low light conditions with lower ETR and NPQ, but higher Fv/Fm and ΦPSII compared to other LI treatments. Furthermore, both ΔF/F’m% and Fv/Fm% recovered faster in 2.4-year-old seedlings than in 6-month-old seedling leaves at high LI conditions from 2 to 30 min of darkness (Fig. 2), indicating that 2.4-year-old seedlings seemed adapted to high LI with higher ΔF/F’m% and Fv/Fm (%) for photo-protection compared to 6-month-old seedlings. In addition, lower ETR and NPQ, but higher Fv/Fm and ΦPSII at low and moderate LI conditions were detected in 2.4-year-old seedlings compared to high LI conditions, suggesting that 6-month-old seedlings favored low PPFD while 2.4-year-old seedlings were adapted to high PPFD. Under dark treatment, protective mechanisms in 2.4-year-old seedlings with higher ΔF/F’m% and Fv/Fm% might prevent their leaves from an excessive reduction in PSII acceptors, avoid excessive energy absorption, and respond with higher PSII photochemical efficiencies (Wong et al. 2014; Lai et al. 2022; Wang et al. 2022).

The 6-month-old seedlings appeared to be sensitive to high light irradiances, as the condition caused serious photo-inhibition and photo-damage. In addition, there were notably lower NPQ, qE, qZ + qT, and qI values in 2.4-year-old seedlings than in 6-month-old seedlings under moderate and high LI conditions, indicating that 2.4-year-old seedlings tended to drive photosynthetic ETR to quenching energy, even when photo-inhibition occurred in these seedlings. The photosynthetic system of 6-month-old seedlings was dominated by qZ in photo-protection, while the photosynthetic system of 2.4-year-old seedlings was more productive and had greater photo-protective ability (Demmig-Adams et al. 2020; Wang et al. 2022). The 2.4-year-old seedlings had a lower qE, and the xanthophyll cycle also maintained the same proportion. The increase in the xanthophyll cycle of NPQ is caused by the change in the structure of the PSII antenna system; that is, the change in the rate of heat dissipation of excess light energy (La Porta et al. 2005). Figure 3 shows that the ratio of qZ + qT in 6-month-pld seedlings increased under 300 μmol m^−2^ s^−1^ PPFD, whereas the ratio of qZ + qT in 2.4-year-old seedlings increased under 1000 μmol m^−2^ s^−1^ PPFD, suggesting that 2.4-year-old seedlings could adapt to higher luminosity. In addition, Fig. 4B and D also show that the photo-inhibition of 2.4-year-old seedlings was low under 1000 ~ 2000 μmol m^−2^ s^−1^ PPFD. Consequently, we speculate that 2.4-year-old seedlings were more efficient in the excess energy dissipation. Higher NPQ is a protective mechanism from the damage of photo-inhibition and photo-oxidation (Feng et al. 2002). The fastest and most important component of NPQ is qE, whereas the slowest reaction component of NPQ is qI related to photo-inhibition or slow reversible recovery of the PSII reaction center. After the completion of reaction in the subunit PsbS, zeaxanthin is combined with PsbS protein to dissipate H^+^, and qT (phosphorylation shift-dependent quenching) shows the phosphorylation shift of LHC between PSII and PSI (Nilkens et al. 2010). Excess light energy produced photo-inhibition, and qI was higher in 6-month-old than in 2.4-year-old seedlings (Fig. 3). The higher the qE value was, the stronger the photo-protection mechanism. Higher ΦPSII was detected under low LI conditions with lower qE and qI values, and photo-inhibition% decreased as well, whereas qE and qI values increased as ΦPSII decreased and photo-inhibition% increased under high LI treatments (Fig. 4). A similar result was also observed in Lai et al. (2022) experiments on the range of light adaptation in the habitats of *Eulophia dentata*, *Bletilla formosana*, and *Saccharum spontaneum*. Additionally, a significant and negative relationship was detected between qE and photo-inhibition%, but a significant and positive relationship was detected between qI and photo-inhibition% in all seedlings (Fig. 4), indicating that the path of energy flow to qI was used mainly for photo-inhibition at this stage, and 2.4-year-old seedlings may remain photo-chemically active and able to maintain lower qI under high illumination. The 2.4-year-old field-grown seedlings had high photo-protection ability at high LI conditions, and these seedlings may adjust the path of energy flow absorption using heat quenching. During the photosynthesis period, changes in photo-inhibition were mainly affected by qI and qE, followed by maintaining photo-protection. These results demonstrate that the larger the qE, the lower the photo-inhibition, and the higher the qI, the higher the photo-inhibition. Simple evaluations of photosynthesis can be made and relationships between heat quenching and photosynthetic efficiency can also be estimated, and variable photosynthesis parameters are highly sensitive indicators representing the physiological status of tested plants, providing a quick means to identify the physiological condition of plants (Wang et al. 2022). Therefore, qE and qI can be used as indicators of photo-protection and photo-inhibition, respectively. These results might be useful in efforts to predict photosynthetic responses to light induction in different seedling ages of* M. oiwakensis.*

The susceptibility of photosynthesis to photo-inhibition strongly depends on LI. Under high irradiance, the light reaction can absorb more photons than can be used for carbon fixation reactions; e.g., by leaves in the upper canopy layer exposed to the sun and also by shade leaves exposed to sunflecks (D’Ambrosio et al. 2006). Stress decreases the ability of photosynthetic systems to utilize incident photons, thus leading to photo-inhibition and reduced quantum yields of photochemistry and ChlF. Conversely, under low LI that are limiting to photosynthesis, zeaxanthin is converted to violaxanthin, and the reverse reaction occurs at high LI that exceed the level of light that can be consumed by photochemistry (Demmig-Adams et al. 2020). ETR is the product of PSII efficiency, absorbed light, and the relative rate of electron transport through PSII. Thus, the elevated ETR, ΔF/F’m, and Fv/Fm level of 2.4-year-old seedlings may help plants avoid high-illumination damage from excess energy. Electrons transferred from PSII to PSI are used by downstream electron sink pathways, including photosynthetic carbon fixation and photorespiration. When carbon fixation becomes saturated, photosynthesis is unable to use all of the energy absorbed by plants under high LI conditions (Dewir et al. 2015). The increased allocation of excitation energy to photorespiration can effectively maintain linear photosynthetic electron transport and appropriately utilize excitation energy for CO_2_ assimilation under photo-inhibition, thus alleviating photo-damage (Jiang et al. 2006). In our study, as LI increased, the higher ETR values of 2.4-year-old seedlings tended to result in a higher photosynthetic efficiency relative to 6-month-old seedlings, suggesting that 2.4-year-old seedlings have adapted to high LI conditions and exhibited higher values of absorbed light utilized in photosynthesis than 6-month-old seedlings under high LI conditions. Our results provide a theoretical basis for afforestation in *Mahonia* plantations using native species. Since qE or qI is easy to measure in open field, and provides a useful indicator for *Mahonia* species restoration, habitat creation, construction, and ecological monitoring by judging whether a plant is in a suitable luminosity cultivation environment or in a high luminosity stress.

## Conclusions

The 6-month-old and 2.4-year-old seedlings of *M. oiwakensis* growing in subtropical regions display different capacities adapted their photosynthesis to low LI and high LI, respectively, and exhibiting protective mechanisms to avoid damage to the photosynthetic apparatus. ETR, ΔF/F’m%, and Fv/Fm% of 2.4-year-old seedlings were elevated under high LI, whereas higher NPQ levels of 6-month-old seedlings were detected under low LI compared to 2.4-year-old seedlings. The qI increased and qE decreased as photo-inhibition% increased, and higher photo-inhibition% was observed in 6-month-old seedlings compared to 2.4-year-old seedlings under high LI treatments. An optimal strategy of LI regulation will help in designing growth chambers and greenhouse light environments to grow these seedlings, and can be useful for predicting the changes in performance and distribution of the seedlings for *M. oiwakensis*.

## Supplementary Information

**Additional file 1: Fig. S1.** *Mahonia oiwakensis* 6-month (panel A) and 2.4-year (panel B) seedlings. **Fig. S2.** Monthly air temperatures (bar), precipitation (circle), and photosynthetic photon flux density (PPFD, less than 100 μmol m^–2^ s^–1^) during the study period from January to December 2021 at the Nantou Mountain area of Taiwan (23°38'54.7"N 120°47'40.6"E). **Fig. S3.** Light response curve in electron transport rate (ETR, panel A), non-photochemical quenching (NPQ, panel B), and both Fv/Fm and PSII efficiency (ФPSII, panel C) for 6-month and 2.4-year old seedlings of *Mahonia oiwakensis*. Plants were measured under 0, 35, 60, 90, 120, 175, 260, 400, 600, 900, 800, 1,200, 1,700 and 2,100 μmol m^-2^ s^-1^. Error bar = standard error, n = 5.

## Data Availability

The datasets used and/or analysed during the current study are available from the corresponding author on reasonable request.

## Abbreviations

Chl: Chlorophyll
ChlF: Chlorophyll fluorescence
NPQ: Non-photochemical quenching
PS II: Photosystem II
qE: Energy-dependent quenching
qI: Photo-inhibitory quenching
qT: State transition quenching
qZ: Zeaxanthin-dependent quenching
LHC: Light-harvesting complexes
Fv/Fm: Potential quantum efficiency of PSII
ΔF / Fm: Actual PSII efficiency
Fo: Minimal ChlF
Fm: Maximal ChlF
ETR: Electron transport rate
