# Supplementary material for: Responses of photosynthesis and chlorophyll fluorescence during light induction in different seedling ages of Mahonia oiwakensis
Source: Bot Stud. 2023 Mar 9;64:5. doi: 10.1186/s40529-023-00369-w (PMC9995626; doi:10.1186/s40529-023-00369-w)
Supplement: Supplementary file 1 — Additional file 1: Fig. S1. Mahonia oiwakensis 6-month (panel A) and 2.4-year (panel B) seedlings. Fig. S2. Monthly air temperatures (bar), precipitation (circle), and photosynthetic photon flux density (PPFD, less than 100 μmol m–2 s–1) during the study period from January to December 2021 at the Nantou Mountain area of Taiwan (23°38'54.7"N 120°47'40.6"E). Fig. S3. Light response curve in electron transport rate (ETR, panel A), non-photochemical quenching (NPQ, panel B), and both Fv/Fm and PSII efficiency (ФPSII, panel C) for 6-month and 2.4-year old seedlings of Mahonia oiwakensis. Plants were measured under 0, 35, 60, 90, 120, 175, 260, 400, 600, 900, 800, 1,200, 1,700 and 2,100 μmol m-2 s-1. Error bar = standard error, n = 5. [file 40529_2023_369_MOESM1_ESM.doc]

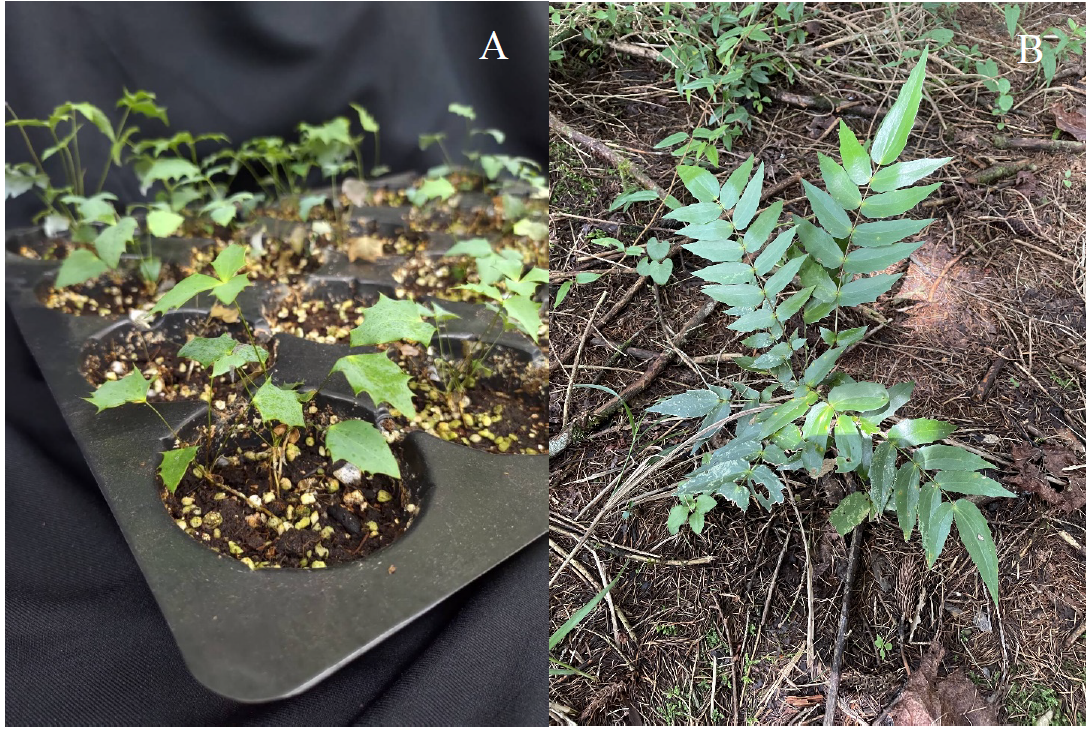


Additional file 1: Fig. S1. *Mahonia oiwakensis* 6-month (panel A) and 2.4-year (panel B) seedlings.

Additional file 1:Fig. S2. Monthly air temperatures (bar), precipitation (circle), and photosynthetic photon flux density (PPFD, less than 100 μmol m^–2^ s^–1^) during the study period from January to December 2021 at the Nantou Mountain area of Taiwan (23°38'54.7"N 120°47'40.6"E).

Additional file 1:Fig. S3. Light response curve in electron transport rate (ETR, panel A), non-photochemical quenching (NPQ, panel B), and both Fv/Fm and PSII efficiency (ФPSII, panel C) for 6-month and 2.4-year old seedlings of *Mahonia oiwakensis*. Plants were measured under 0, 35, 60, 90, 120, 175, 260, 400, 600, 900, 800, 1,200, 1,700 and 2,100 μmol m^-2^ s^-1^. Error bar = standard error, n = 5.
